# Microstructural Inhomogeneity in the Fusion Zone of Laser Welds

**DOI:** 10.3390/ma16217053

**Published:** 2023-11-06

**Authors:** Libo Wang, Xiuquan Ma, Gaoyang Mi, Lei Su, Zhengwu Zhu

**Affiliations:** 1 The State Key Laboratory of Digital Manufacturing Equipment and Technology, School of Mechanical Science and Engineering, Huazhong University of Science and Technology, Wuhan 430074, China; wlb@hust.edu.cn (L.W.);; 2 Optics Valley Laboratory, Ezhou 430074, China; 3 Guangdong Intelligent Robotics Institute, Dongguan 523808, China; 4 The State Key Laboratory of Material Processing and Engineering, School of Materials Science and Engineering, Huazhong University of Science and Technology, Wuhan 430074, China

**Keywords:** aluminum alloy, laser welding, microstructure, crystal orientation

## Abstract

This paper investigated evolutions of α-Al sub-grains’ morphology and crystalline orientation in the fusion zone during laser welding of 2A12 aluminum alloys. Based on this, a new method for assessing the weldability of materials was proposed. In laser deep-penetration welding, in addition to the conventional columnar and equiaxed dendrites, there also exhibited a corrugated structure with several ‘fine-coarse-fine’ transformations. In such regions, an abnormal α-Al coarsening phenomenon was encountered, with a more dispersed crystalline orientation arrangement and a decreased maximum pole density value. Particularly, structural alterations appeared more frequently in the weld bottom than the top. The above results indicated that the laser-induced keyhole presented a continually fluctuating state. Under such a condition, the solid–liquid transformation exhibited an unstable solidification front, a fluctuant undercooling, and a variational solidification rate. Meanwhile, the welding quality of this material is in a critical state to generate pores. Therefore, the appearance and relevant number of corrugated regions can be considered as a new way for judging the weldability, which will help to narrow the processing window with better welding stability.

## 1. Introduction

As 2000-series aluminum alloys exhibit high specific strength and excellent plasticity, and are easy to process, they are widely applied in various high-load components and structures, such as aircraft skins, frames, rib sections, and wing beams [1]. As designed, they comprised minor amounts of copper (ranging from 3.8% to 4.9%) and magnesium (ranging from 1.2% to 1.8%), which commonly formed low melting point phases, namely Al_2_CuMg (S phase) and Al_2_Cu (θ phase) [2,3,4]. During laser welding, the existence of low melting point phases blocks the continuity of the liquid film distribution on the front surface of the keyhole. Upon laser irradiation, these aforementioned phases will instantaneously undergo temperature elevation and vaporization, leading to oscillation and collapse of the keyhole during the progression of the laser. Under such conditions. porosity and cracks were frequently encountered [5,6,7].

However, with the high welding speed and energy density in laser welding, the solidification process was complicated by the rapid melting and solidification of the material, as well as the melt flow and keyhole disturbance in the molten pool [8,9,10]. In previous research, the welding solidification process has been found to directly affect the morphology and dimensions of the weld’s microstructure [11,12]. Therefore, understanding the solidification characteristics of the molten pool and the distribution patterns of grains during the welding process was of paramount importance. Extensive investigations have been conducted by employing various techniques, including arc welding [13,14,15], laser welding [16,17,18], and friction stir welding [19,20,21]. Generally, the evolution of the weld solidification mode can be comprehended through the classical theory of constitutional undercooling in the field of solidification [22]. From the fusion boundary to the centerline, the mode transforms from planar into cellular, columnar, and equiaxed growth [23,24,25]. For the weld joint, different solidification modes significantly influenced its final performance. Finer weld microstructures led to improved mechanical properties and better weldability of the material [26,27]. The traditional material weldability analysis standards were mainly consisted of macro observations of joint morphology (millimeter) and internal defects (porosity and inclusions), measurement of mechanical properties, etc. [28,29,30]. However, as a result of the differences between the suppliers of materials, production batches, etc., as well as laser equipment and environmental differences, the welding process was commonly unstable but without apparent defects, exhibiting weak adaptability of the welding process to the unpredictable laser emission, material supply, and environments [12,31].

In this paper, in view of the relatively weak tolerance of the current welding process window to various laser equipment, materials, and environments, evolutions of α-Al sub-grains’ morphology and crystal orientation during the solidification process of 2A12 aluminum alloy welding were investigated. This study focused on the mechanism of the formation of microstructural non-uniformity in the fusion zone of welding joints. Based on this, a new method for assessing the weldability of materials was proposed. This had significant importance in gaining insights into the solidification principles of weld seams, optimizing welding processes, and enhancing the stability of the aluminum alloy welding process.

## 2. Experimental Procedure

### 2.1. Experimental Materials

**Figure 1:**
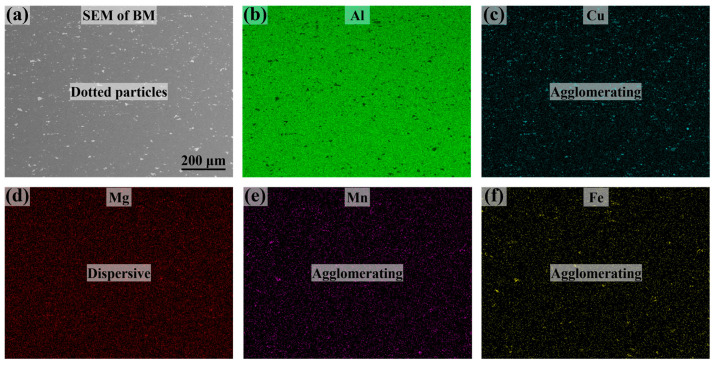
Cross-sectional morphology and major composition profiles of the 2A12 aluminum alloy. (**a**) Dispersed secondary phase particles in the BM; (**b**–**f**) mappings of the Al, Cu, Mg, Mn, and Fe elements.

This experiment was performed with the 2A12 aluminum alloy, which was mainly composed of elements, including Al, Mg, Mn, and other minor elements. The specific composition of the base material (BM) is presented in Table 1. The Al matrix was characterized by the presence of dispersed white particle phases, as depicted in the cross-sectional microstructure (Figure 1a).

As displayed in the elemental surface distribution diagram, β-Al (Mg_5_Al_8_) was dispersed in the Al matrix (Figure 1d) [32], while the aggregated particulate phases were Al_2_Cu (Figure 1c) and FeMnAl_6_/FeMn (Figure 1e,f) [33]. Under these compositional conditions, during the process of melting and solidification, the 2A12 aluminum alloy weld was able form a typical microstructure characterized by the presence of α-Al sub-grains, following a dendrite growth model similar to the one proposed by Kurz [34]. Specifically, the Al grains, delineated via grain boundaries, were composed of numerous clusters of α-Al sub-grains, with a minor presence of impurities and second phases distributed between the sub-grains and grain boundaries.

### 2.2. Experimental Methods

Prior to laser welding, the sample surface was prepared by removing the oxide film using a steel wire ball and cleaning the workpiece surface from oil contamination using an acetone solution. The laser welding process utilized the YSM-2000 fiber laser manufactured by Guangdong Guozhi Laser Technology Co., Ltd. (Guangzhou, China). It had a maximum output power of 2000 W, operated at a wavelength of 1080 nm, and had a 14 μm fiber core diameter. During the experiment, to prevent damage to the laser caused by high beam reflections, the laser beam was deflected at a certain angle of 8° for the vertical direction. The morphology of the α-Al sub-grains in the solidification process exhibited different forms based on the degree of undercooling of the solid/liquid interface (S/L) and the fluctuation state of the S/L [22]. Therefore, by analyzing the microstructure in the fusion zone, it was possible to infer the corresponding local solidification conditions to a certain extent.

Based on this, the present study conducted a microscopic analysis of the fusion zone in the welded joint, focusing on the structural morphology and crystal orientation characteristics. Firstly, samples were extracted from the middle part of the weld and subjected to rough grinding, fine grinding, and electrolytic polishing to obtain a smooth cross-sectional surface. The cross-sectional microstructure of the fusion zone was analyzed using scanning electron microscopy (SEM), energy-dispersive spectroscopy (EDS), and electron backscattered diffraction (EBSD). Under the aforementioned conditions, the macro/microstructural features of the fusion zone in the laser-welded joint of the 2A12 aluminum alloy were investigated.

## 3. Results and Discussion

This section has been divided by subheadings. It should provide a concise and precise description of the experimental results, their interpretation, as well as the experimental conclusions that can be drawn.

### 3.1. Characteristics of Welded Joint Formation

**Figure 2:**
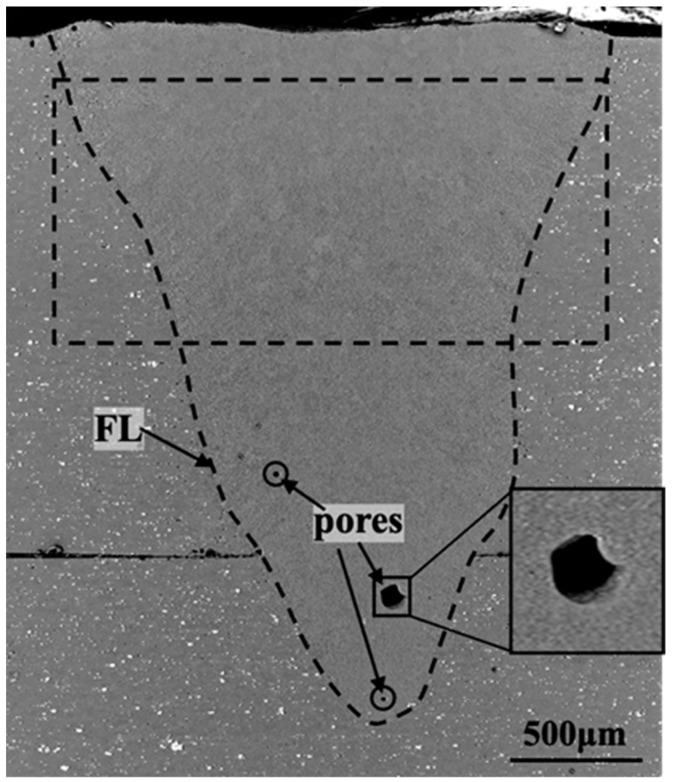
Cross-sectional morphology features of the weld bead, precipitation-type pore (round frame), and process-type pore (square).

Figure 2 depicts a typical morphology of a deep V-shaped laser-welded joint, wherein the penetration depth was measured as 2.60 mm and the melt width spanned 2.13 mm. Given that the aspect ratio was 1.22, which was significantly greater than the 0.5 formed via the heat conduction welding, it was evident that the formation of this weld resulted in the generation of a keyhole.

Notably, the bottom region of the weld exhibited the presence of pores, a prevailing occurrence in aluminum alloy laser welding. These pores could be categorized into two types based on their size and morphology: process pores (indicated by square frames in Figure 2) and precipitation pores (indicated by circular frames in Figure 2). Additionally, the fusion line (FL) located at the bottom of the fusion zone was characterized by a non-horizontal symmetry. These two phenomena indicated that the interaction between the keyhole and the molten pool at the bottom position was more drastic, random, and unstable in comparison to the upper region during the laser welding process, which was prone to cause abnormal fluctuations in the keyhole, bubble motion, and fusion line (initial solidification front).

**Figure 3:**
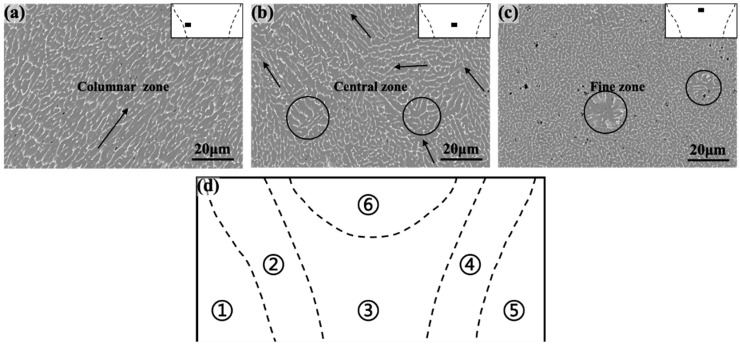
Microstructure and classification of the fusion zone. The squares show the columnar zone (**a**) near the fusion line; the central zone (**b**) in the center of the weld; and the fine zone (**c**) in the upper surface; (**d**) subregions: the BM (① and ⑤), columnar (② and ④), center (③), and fine grain (⑥); where the circles represent locally anisotropic equiaxial crystals.

To delve into the solidification characteristics of the molten pool further, a microstructure analysis was performed on the region enclosed by the dashed box in Figure 2, as depicted in Figure 3. The grizzly in Figure 3 depicts the α-Al sub-grains and the white represents the intergranular phase. Based on the morphology and arrangement of the α-Al sub-grains, three distinct regions were identified:

(a) The columnar zone (Figure 3a), positioned near the fusion line, exhibited elongated columnar structures, with significant length-to-width ratios. The arrows in the columnar zone represented the projection of the growth direction of the α-Al sub-grains onto the cross-section. It was observed that the sub-grains in this region displayed minimal deviation in this direction, indicating that the molten pool in this region experienced high, stable solidification conditions, with a consistent direction towards the maximum temperature gradient (G).
(b) The central zone (Figure 3b), situated in proximity to the center of the weld, encompassed a disorderly mixture of long, short, fine, and coarse α-Al sub-grains. The mixture grains’ region served as a transitional region between the cellular and fine grain regions, and the structural features of α-Al sub-grains in this region consisted of cellular clusters with heterogeneous growth directions (shown by arrows in Figure 3b) and locally anisotropic equiaxed grains (shown by circles in Figure 3b).
(c) The fine grain zone (Figure 3c), located near the upper surface, comprised fine grains exhibiting isotropic growth. In this region, the spacing between α-Al sub-grains significantly decreased, and a few larger equiaxed grains were also observed (circles in Figure 3c).

According to the above microstructural characteristics, the 2A12 aluminum alloy joints could be divided into distinct regions: the BM (① and ⑤), columnar (② and ④), center (③), and fine grain (⑥) zones, as illustrated in Figure 3d.

### 3.2. Weld Macroscopic Grain Distribution Patterns

**Figure 4:**
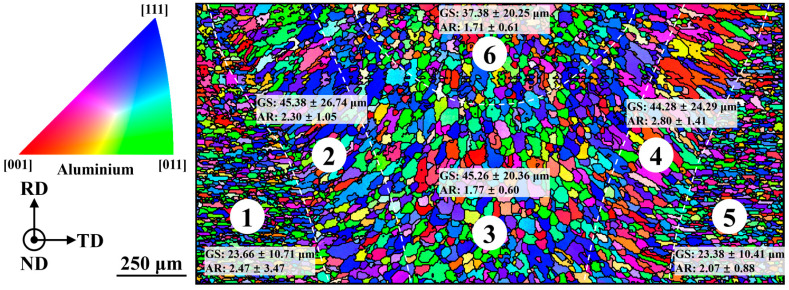
IPF-ND crystalline orientation figures and statistical distributions of the upper FZ.

A quantitative analysis was conducted to examine the crystal growth and orientation patterns of the six regions depicted in Figure 3, specifically focusing on the macroscopic grain features composed of α-Al sub-grain clusters. The resulting inverse pole figure-normal direction (IPF-ND) orientation distribution maps are presented in Figure 4.

From an examination of the grain morphology, it was observed that the grains in the parent material (① and ⑤) exhibited a flattened distribution along the transverse direction (TD), indicative of the typical rolling process along the rolling direction (RD). In the columnar zone (② and ④), the grains displayed preferential growth perpendicular to the fusion line, with symmetrical growth observed on both sides. The grains in the central zone (③) continued the growth direction in the cellular region, which generally showed isotropic equiaxed crystal growth. In the fine grain zone (⑥), the grains demonstrated significant refinement, resulting in a notable reduction in both grain size (GS) and aspect ratio (AR).

Analyzing the GS and AR of the grains in the different regions of the fusion zone, as depicted in Figure 4, resulted in the emergence of several key findings. It could be seen that the GS in the BM was the smallest, approximately 23 μm, owing to the effects of the rolling process, which made the direction of the long axis of the grains parallel to the TD. In the columnar zone, the grains underwent gradual coarsening to around 45 μm through a “competition and elimination” mechanism [22], growing perpendicularly to the fusion line. In the central zone, the GS remained similar to that of the cellular region, but its AR was drastically reduced and its isotropy of growth was enhanced, consistent with the changes in grain morphology in Figure 4. The grains were maintained in the fine grain zone, with equiaxial growth and further refinement to about 37 μm.

**Figure 5:**
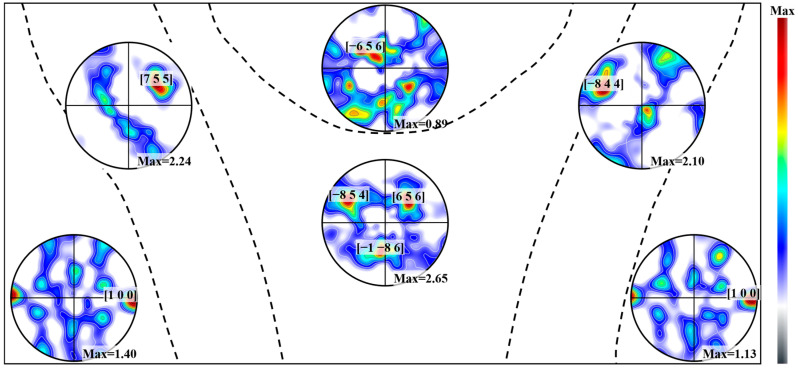
{1 0 0} pole figures and statistical distributions of each subregion in the FZ.

Moreover, the crystal orientation and distribution density of the grains in each partition were further quantitatively analyzed, as shown in Figure 5. Due to the face-centered cubic arrangement of the lattice sites of the atoms in the Al crystals, the <1 0 0> crystal orientation represents the preferred growth direction of α-Al sub-grains. Consequently, the {1 0 0} polar plots were separately plotted for each region, where the spatial coordinates [x, y, z] of the highest density points (representing [1 0 0] crystal orientations) were marked on the map. To eliminate the influence of different test area sizes on the extreme density values observed in the pole maps, the ratio of the area of each zone to that of zone 3 (the largest area) in Figure 4 was calculated by taking zone 3 as a unit of “1”, which was subsequently multiplied by the original maximum pole density in the pole map to obtain the “normalized” pole density value.

Based on Figure 5, it was observed that in the BM, the [1 0 0] crystal orientation was aligned parallel to the TD and perpendicular to the RD. In the columnar region, the [7 5 5] and [−8 4 4] orientations represented the symmetric growth of [1 0 0] crystals on both sides of the fusion line, exhibiting an increased growth concentration (1.40→2.24, 1.13→2.10). In the central region, this growth became more diverse, with the [6 5 6] and [−8 5 4] orientations following the growth patterns observed in the cellular zones on the left and right sides, respectively. In the fine grain region, there was a significant decrease in the maximum pole density value. In conjunction with the microstructural distribution depicted in Figure 3a–c, an intrinsic correlation existed between the α-Al sub-grains and the grains formed by clusters; essentially, each grain was gradually formed through the homogeneous epitaxial growth of the initial individual α-Al sub-grains.

### 3.3. Weld Macroscopic Grain Distribution Patterns

**Figure 6:**
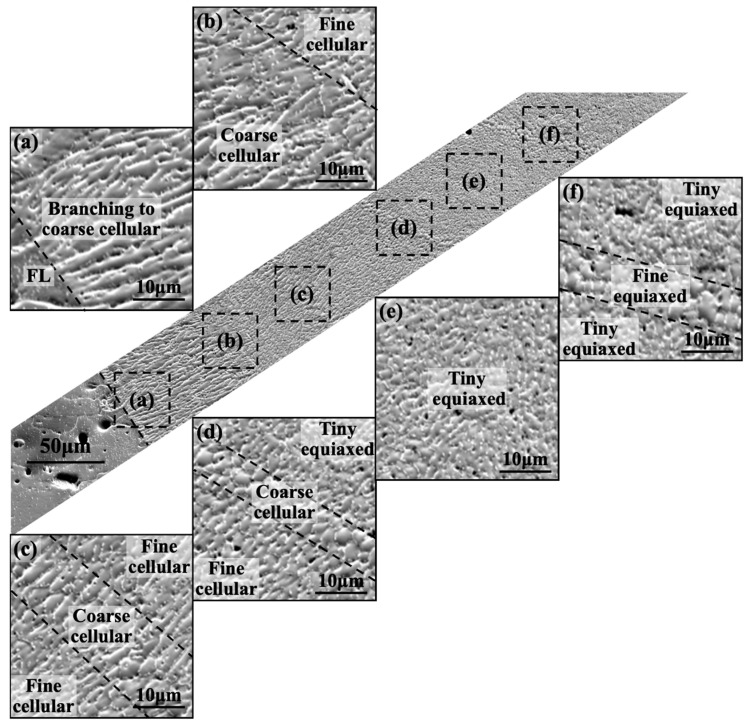
Microstructure evolution of α-Al sub-grains from the BM to the weld center. (**a**) Tip branching and coarsening near the FL; (**b**) cellular refinement; (**c**) fine-coarse-fine transformation in the cellular zone; (**d**) cellular coarsening refinement and changed to tiny equiaxed; (**e**) tiny equiaxed; and (**f**) fine-coarse-fine transformation in the equiaxed.

In order to investigate the reasons behind the crystal orientation and size variations along the path of regions 1→2→3→6 (equivalent to 5→4→3→6) in Figure 4, the microstructural evolution of α-Al sub-grain along this path was examined, as shown in Figure 6. Overall, from the fusion line to the center of the weld, the α-Al sub-grains underwent a morphological transition from cellular to equiaxed and showed several transitions of alternating between coarseness and fineness in the transverse dimensions, giving rise to a visually distinctive corrugated structure. Near the fusion line (Figure 6a), after an extremely narrow planar growth, the α-Al sub-grains rapidly branched, formed tips, and eventually transformed into larger cellular α-Al sub-grains. Subsequently, a smooth transition occurred, leading to smaller cellular sub-grains (Figure 6b). This was followed by a sequence of transitions between fine-coarse-fine cellular structures (Figure 6c). Furthermore, the cellular sub-grains initially coarsened and then refined into equiaxed structures (Figure 6d), gradually evolving into equiaxed sub-grains (Figure 6e). In the center of the weld, a similar small-large-small equiaxed structure transition was observed in the α-Al sub-grains.

Based on the above results, it was observed that the solidification process from the fusion line to the center of the weld exhibited localized, non-uniform, and discontinuous characteristics. In this process, in the vicinity of the advancing S/L, the temperature, solute, and flow fields underwent intense fluctuations, leading to the disruption of the continuity in the distribution of G, the solidification rate (R), and undercooling. Under typical near-equilibrium or quasi-steady-state solidification conditions, from the fusion line to the center of the weld, the G at the S/L gradually decreased, while the R increased. As a result, the G/R ratio decreased, causing an increase in undercooling and the nucleation rate, leading to the formation of a greater number of α-Al sub-grains. Additionally, the G×R value increased, resulting in a shorter grain growth time and a decrease in the size of the α-Al sub-grains. Following this trend, the α-Al sub-grains in the weld underwent a transition from columnar (or cellular) growth to equiaxed growth, accompanied by a refinement in their microstructural size (Figure 6). However, under conditions of discontinuous solidification, the shape and size of α-Al sub-grains in the weld exhibited fluctuations. Furthermore, the closer the location was to the center of the weld, the more pronounced the microstructural variations caused by these fluctuations became (Figure 6a,b).

**Figure 7:**
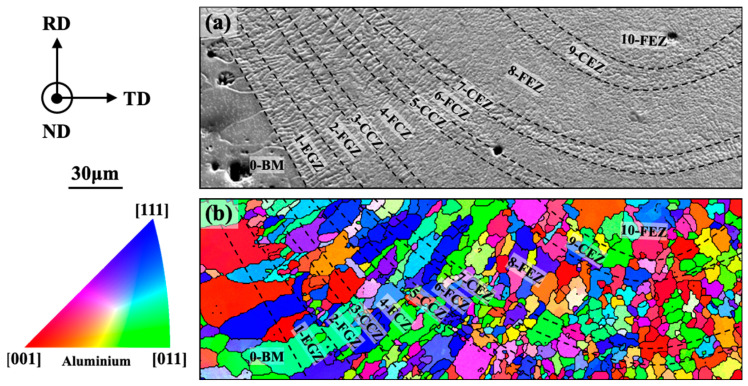
Evolutions of microstructure and crystalline orientation at the weld bottom. (**a**) Morphology of α-Al sub-grains; and (**b**) orientation map of IPF-ND.

A further investigation was conducted into the microstructural and orientation evolution near the bottom of the weld. From the fusion line to the center of the weld, the α-Al sub-grains underwent the following morphological changes (Figure 7a): they originated from the BM and progressed through the epitaxial growth zone (EGZ) → the free growth zone (FGZ) → the coarse cellular zone (CCZ) → the fine cellular zone (FCZ) → CCZ → FCZ → the coarse equiaxed zone (CEZ) → the fine equiaxed zone (FEZ), and finally the CEZ and FEZ. These results indicated that similar microstructural evolutions occurred at the bottom of the weld, with the transitions between the cellular and equiaxed zones being more frequent, indicating more pronounced fluctuations in the temperature, solute, and flow fields during the solidification process near the bottom. In Figure 7b, an IPF was generated based on the ND direction as a reference projection for the orientation distribution, where solid black lines indicate grain boundaries. The map revealed the presence of newly formed grains near the fusion line (1-EGZ → 2-FGZ), in the intermediate region (4-FCZ → 5-CCZ), and the central region (8-FEZ → 9-CEZ). These grain transitions resulted in a refinement of GS from the BM to the center of the weld.

**Figure 8:**
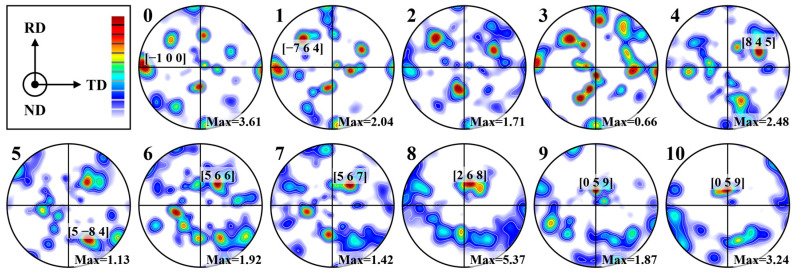
{1 0 0} pole figures and pole point arrangement of α-Al sub-grains at the weld bottom.

The growth characteristics of the grains in Figure 7 were further quantitatively analyzed by plotting {1 0 0} pole maps for each of the 11 regions, as presented in Figure 8. The spatial coordinates [x, y, z] of the highest density point ([1 0 0] crystal orientation) in each pole map were indicated in this figure. According to the previously mentioned method, the ratio of the area of each zone to that of zone 8 (the largest area) in Figure 8 was calculated by taking zone 8 as a unit of “1”, which was then multiplied by the original maximum pole density in the pole map to obtain the “normalized” pole density value.

In the BM, the [1 0 0] crystal orientation was parallel to the TD and perpendicular to the RD, exhibiting a typical rolling texture that was consistent with the results shown in Figure 5. In the EGZ, the distribution of the [1 0 0] crystal orientation was essentially similar to that in the BM, indicating a more synergistic epitaxial growth of α-Al sub-grains in terms of both their morphology and orientation. Subsequently, the “competition and elimination” mechanism between the newly formed and pre-existing α-Al sub-grains significantly diminished the presence of the rolling texture. The decrease in the maximum pole density values (from 2.04 to 1.71 to 0.66) indicated a diversification of growth directions for α-Al sub-grains, and the movement of the maximum pole density point indicated a change in the preferred growth direction (from [8 4 5] to [5 −8 4]). Finally, the growth direction of the α-Al sub-grains gradually approached [0 5 9], with a decreasing angle relative to the laser welding direction. Based on the analysis presented in Figure 7a, the corrugated structure of α-Al sub-grains was mainly observed in zones 3, 5, 7, and 9. It was observed that these four regions exhibited a more dispersed pole distribution and smaller maximum pole density values compared to their adjacent regions. These results indicated that such fluctuations resulted in the formation of coarser α-Al sub-grains in terms of their structure and a more dispersed growth pattern in terms of their orientation.

### 3.4. Mechanisms for the Formation of Non-Uniform Organizations

For the complete grains formed by the α-Al sub-grain clusters, differences in their grain morphology were observed between the columnar zone and the center zone of the weld, while their sizes were almost close to each other (Figure 4). The grain growth directions were notably different, but the degree of grain clustering was generally consistent (Figure 5), and there was no manifestation of common continuous growth and coarse grains. In contrast, the GS in the fine grain zone was noticeably refined, and the degree of grain clustering was significantly reduced.

#### 3.4.1. Equiaxed Crystals in the Central and Fine Grain Zone

For the center zone, from the fusion line to the center of the weld, the G gradually decreased, while the R progressively increased, leading to an increase in undercooling. At the S/L, a large number of high-melting-point nuclei initially precipitated. Through the constraint of lattice matching, α-Al sub-grains underwent heteroepitaxial growth around the nuclei, resulting in the coexistence of cellular grains, with random growth directions and equiaxed grains with isotropic growth in the mixed region.

For the fine grain zone, during the welding process, the high temperature of the keyhole’s wall caused by the laser resulted in a significantly higher temperature at the front of the melt pool compared to the rear. This led to the backward flow of the molten pool due to surface tension. Additionally, the low-melting-point phase at the keyhole’s wall easily melted or even sublimated due to its high laser absorption rate, effectively “purifying” the adjacent aluminum alloy melt. In both of these cases, a significant G was formed due to the “rapid cooling” effect of the air near the surface, resulting in a solidification transition that exhibited characteristics similar to “deep undercooling”. Moreover, the strong G, coupled with convection in the melt pool and the descent of “crystal nuclei rain” on the melt pool surface, promoted growth and coarsening of the grains, leading to the formation of equiaxed grain structures with significant differences in their sub-grain spacing.

#### 3.4.2. Corrugated Organization of the Columnar Region

In the casting and arc welding processes of the 2A12 aluminum alloy, the temperature and solute fields in the melt were relatively ordered and smooth, allowing for a steady advancement in the S/L interface during the solidification process [35]. There were no significant temperature and solute fluctuations, resulting in a consistent and uniform solidification structure and size. When reaching the critical point of undercooling transition (determined via G and R), distinct microstructural transitions between planar, cellular, columnar, and equiaxed grains were observed, consistent with the findings of Kurz’s research [31]. Based on the solidification characteristics of the laser welding molten pool, there were temporal and spatial differences in solidification between the columnar zone and the center zone. In the former, the bottom of the weld solidified before the top, and the sides of the weld solidified before the center. In the latter, the bottom of the weld was closer to the keyhole than the top, and the sides of the weld were closer to the keyhole than the center. According to the metallurgical theory of laser welding small molten pools, in the same horizontal plane, the regions that solidified first had higher G and smaller R values, resulting in lower undercooling rates and predominantly epitaxial growth. The regions that solidified later had lower G and larger R values, resulting in higher undercooling rates and predominantly free nucleation growth. Therefore, from the fusion line to the center of the weld, the α-Al sub-grains exhibited a transition of epitaxial growth, competitive elimination, and free nucleation, leading to the formation of cellular grains with specific growth directions (Figure 4, region 2) and disordered growth of equiaxed grains (Figure 4, region 3). Consequently, both the top and bottom of the weld exhibited this structural and orientation transition in accordance with the overall pattern.

However, in laser deep penetration welding, the high-power density laser beam interacts with the low melting point aluminum alloy, resulting in the formation of a significant amount of metal vapor at the beam spot. Under the recoil pressure of the metal vapor, a keyhole was formed. The energy input of the laser at the beam’s waist and the distribution of material composition and phases in the aluminum alloy made it difficult to achieve complete uniformity and consistency. As a result, the shape of the keyhole’s inner wall determined the Fresnel absorption efficiency of the laser [36]. Therefore, the longitudinal depth, opening diameter, and curvature of the front and rear walls of the optically induced keyhole were in a state of random variation. The rapid and dynamic fluctuations in the keyhole’s inner wall directly transfer to the molten pool in contact with it at the rear and propagate to the distant solidification boundary. Moreover, the number of microstructural transitions in the top region of the weld (Figure 6) was fewer, and the intervals were longer compared to the bottom region of the weld (Figure 7). Thus, the closer the region was to the keyhole, the stronger the effect of the oscillations on the molten metal, leading to significant variations in the original solute and temperature distributions at the S/L, making it more prone to instability.

**Figure 9:**
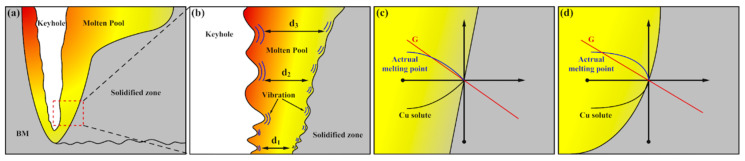
Schematic diagram of the keyhole oscillation principle in the fusion zone. (**a**) The distribution of keyhole, molten pool and solidification region; (**b**) oscillating impact energy transfer, where d1, d2, d3 represent the distance between the back wall of the keyhole and the solidification boundary; (**c**,**d**) undercooling at the S/L.

Based on the laser deep penetration welding process, the distribution of the keyhole, molten pool, and solidification region is shown in Figure 9a. Influenced by the laser energy and material distribution uniformity, the keyhole was in a state of constant oscillation and variation. The impact energy generated via the oscillations was transmitted between the rear wall of the keyhole and the solidification boundary (Figure 9b). Furthermore, the bottom region had a shorter transmission distance compared to the upper regions (d3 > d2 > d1), resulting in less energy loss and higher intensity and frequency of oscillations at the bottom.

Within one oscillation cycle of the keyhole, the molten metal from the distant region, with a higher temperature and lower solute concentration, was transported towards the vicinity of the solidification interface. As a result, it led to the following findings: (1) The oscillation effect caused the temperature gradient at the S/L interface to decrease, which led to an increase in the degree of undercooling. (2) The transfer of the low-solute concentration melt near the keyhole to the S/L interface, with a solute concentration difference at the oscillation, resulted in a compositional undercooling effect. (3) The oscillation effect resulted in the melting of the incipient dendritic arms at the S/L interface and an increase in the nucleation undercooling (Figure 9c,d). Additionally, under a nearly constant R, a decreased G resulted in a lower value, leading to a coarsening of the α-Al dendrites (increased primary spacing). Based on these effects, the initial continuous or uniform microstructure underwent a sudden transition, and after the oscillations ceased, it returned to the original solidification state (Figure 9c,d).

In comparison, the central region and fine grain region of the laser deep penetration weld in the 2A12 aluminum alloy were situated at a greater distance from the keyhole, experiencing fewer oscillatory impacts. As a result, their solidification process and microstructure conformed to the conventional size and structural distribution observed in welds. This corrugated characteristic was similarly observed in other laser deep penetration welds exhibiting “dendritic” solidification features [7,37]. The presence of such periodic oscillations indicated that the keyhole was in a continuously fluctuating state, resulting in poor welding process stability, and the welding quality of the material was at a critical state. When the fluctuations of the keyhole intensified further, it collapsed, thereby generating gas pores. Even under conditions where other factors were kept consistent, the inherent material’s non-uniformity introduced significant uncontrollability in welding quality, affecting the material’s ultimate industrial application. 

Using the quantity of this structure as a new criterion for assessing material weldability and further optimizing welding process parameters, monitoring and controlling the formation of the corrugated region could better determine the welding quality. This approach allowed for obtaining a welding process window with improved welding stability and greater tolerance to material and environmental variations. Consequently, it enhanced the reliability and quality of aluminum alloy welding, especially in welding applications that demanded high quality and stability.

## 4. Conclusions

In this paper, a systematic investigation was carried out on the distribution patterns of grains, the evolution of grain orientations, and the mechanism behind the formation of non-uniform microstructures in aluminum alloy laser welding. The specific conclusions were as follows:

- In addition to the conventional structured columnar zone, disordered mixed central zone, and isotropic equiaxed zone, a locally non-uniform and discontinuous corrugated grain structure was observed during the solidification process from the fusion line to the center of the weld for α-Al sub-grains.
- Anomalous α-Al coarsening occurred in these regions, with a more dispersed crystallographic orientation arrangement and a lower maximum pole density value. In particular, the structural changes were more frequent at the bottom of the weld than the top. The reason for their formation was that the laser-induced keyhole showed continuous fluctuation. It destabilized the S/L interface, which caused the fusion of the growing dendritic arms and increased the supercooling at the oscillation to improve the nucleation rate, whereas the decrease in the temperature gradient increased the growth time of the α-Al sub-grains to be coarsened.
- The existence of periodic oscillations indicated that the keyhole was in a continuous fluctuation, and that the weld quality of the material was in a critical state. Even with other consistent conditions, the inherent inhomogeneity of the material could result in a significant uncontrollability in weld quality. The quantity of corrugated areas was used as a new way of determining material weldability (micron level), and monitoring and controlling their formation resulted in a process window with better weld stability and greater tolerance for material and environmental changes.

## Figures and Tables

**Table 1:** Chemical composition of the BM.

Element	Cu	Mg	Mn	Fe	Zn	Al
Wt. %	4.87	1.28	0.61	0.21	0.49	Balance

## Data Availability

Data will be made available on request.
